# Bidirectional lncRNA Transfer between *Cuscuta* Parasites and Their Host Plant

**DOI:** 10.3390/ijms23010561

**Published:** 2022-01-05

**Authors:** Yuguo Wu, Dong Luo, Longfa Fang, Qiang Zhou, Wenxian Liu, Zhipeng Liu

**Affiliations:** State Key Laboratory of Grassland Agro-Ecosystems, Key Laboratory of Grassland Livestock Industry Innovation, Ministry of Agriculture and Rural Affairs, College of Pastoral Agriculture Science and Technology, Lanzhou University, Lanzhou 730020, China; wuyg16@lzu.edu.cn (Y.W.); luod15@lzu.edu.cn (D.L.); fanglf@lzu.edu.cn (L.F.); zhouq2013@lzu.edu.cn (Q.Z.); liuwx@lzu.edu.cn (W.L.)

**Keywords:** *Cuscuta*, host plants, long non-coding RNA transfer, transcriptome sequencing, interaction network

## Abstract

Dodder species (*Cuscuta* spp.) are holoparasites that have extensive material exchange with their host plants through vascular connections. Recent studies on cross-species transfer have provided breakthrough insights, but little is known about the interaction mechanisms of the inter-plant mobile substances in parasitic systems. We sequenced the transcriptomes of dodder growing on soybean hosts to characterize the long non-coding RNA (lncRNA) transfer between the two species, and found that lncRNAs can move in high numbers (365 dodder lncRNAs and 14 soybean lncRNAs) in a bidirectional manner. Reverse transcription-polymerase chain reaction further confirmed that individual lncRNAs were trafficked in the dodder–soybean parasitic system. To reveal the potential functions of mobile transcripts, the Gene Ontology terms of mobile lncRNA target genes were predicted, and mobile dodder target genes were found to be mainly enriched in “metabolic process”, “catalytic activity”, “signaling”, and “response to stimulus” categories, whereas mobile soybean target genes were enriched in organelle-related categories, indicating that specific mobile lncRNAs may be important in regulating dodder parasitism. Our findings reveal that lncRNAs are transferred between dodder and its host soybean plants, which may act as critical regulators to coordinate the host–dodder interaction at the whole parasitic level.

## 1. Introduction

Parasitism represents a lifestyle in which parasitic plants obtain nutrients from hosts, causing serious biotic stresses and impacts on global agriculture [1]. *Cuscuta* spp. (dodder) are rootless and leafless stem parasites throughout their lifecycle, and cannot survive independently due to their very limited or absent photosynthesis. Their wide host range includes vegetables, crops, and pastures, and they are malignant parasitic weeds [2]. The dodder penetrates the host and forms a specific organ—the haustorium—for host attachment; the vascular connections established by the haustoria serve as an open hub for the exchange of various substances (e.g., water, nutrients, pathogens, systemic signals, and even macromolecules) between the two plants [3]. This exchange is known as cross-species transmission.

Given the importance of cross-species transmission for adaptation, interaction, and evolution in parasitic systems, the study of cross-species transmission has become a popular subject. Since the 1960s, researchers have performed many studies on cross-species transmission. For instance, viruses [4,5] and phytoplasmas [6] have long been known to be transferred between hosts and dodder. A large number of proteins have been shown to be transferred between hosts and dodder, and long-distance mobile proteins can even be transferred to the seeds of foreign plants and among dodder bridge-connected hosts [7]. Systemic signals, including salt stress- and herbivory-induced signals, have also been reported to be transmitted from the dodder to the host plant, and even among dodder-connected hosts [8,9,10]. In addition, recent studies on cross-species transmission at the transcription level have provided breakthrough insights into host–parasite interactions. The bidirectional mobility of large-scale mRNAs has been demonstrated between dodders and host plants, providing potential mechanisms for RNA-based interactions in symplastic connections [11,12]. It has been shown that parasite microRNAs (miRNAs) can transfer into host plants and may act as virulence factors of host gene expression to promote the establishment of parasitic relationships [13]. Small interfering RNAs (siRNAs) can also migrate into the parasite, where they decrease the expression of parasite genes, providing great potential for gene-editing-based dodder prevention [14,15]. Despite the progress that has been made in detailing these processes, our understanding of the cross-species transmission and functional effects of non-coding RNAs (ncRNAs), such as long non-coding RNAs (lncRNAs), is still limited.

NcRNAs are a type of RNA that cannot encode proteins, but can still participate in various biological processes, such as cell growth, proliferation, differentiation, and apoptosis [16,17,18,19]. These ncRNAs comprise regulatory and housekeeping ncRNAs, as well as ncRNAs of unknown function; the regulatory ncRNAs can be further sub-divided into several categories, including siRNAs, miRNAs, and lncRNAs, according to their size [20,21]. In general, lncRNAs represent a large class of RNAs having transcripts longer than 200 nucleotides (nt) in length and poor protein-coding potential [22,23]. Early studies questioned the importance of lncRNAs and regarded them as transcriptional “noise” but, at present, many thousands of lncRNAs—transcribed from locations throughout both plant and animal genomes—have been identified by tilling and RNA-seq analyses [24,25,26]. These lncRNAs are classified into long intergenic non-coding RNAs (lincRNAs), intronic lncRNAs, sense, and antisense lncRNAs, according to their relative location with protein-coding genes [27].

Regulatory roles for these lncRNAs in chromatin modification and transcription are currently under intense investigation [24]. Studies have revealed that lncRNAs can coordinate gene expression, through a hormone–redox–cell wall network, to regulate growth process in plants, such as tomato fruit cracking [28]. In *Arabidopsis thaliana* (L.) Heynh., *DROUGHT INDUCED lncRNA* (*DRIR*) regulates the plant response to drought and salt stress as a novel positive regulator [29]. LncRNAs can also participate in other abiotic stress responses in plants, such as heat stress, cold stress, and oxidative stress [30,31,32,33]. A recent study has found that tomato *lncRNA23468* modulated the accumulation of *NBS-LRRs* in the interaction between *Phytophthora infestans* (Mont.) de Bary and tomato by decoying the expression of *miR482b*, indicating that lncRNAs can also respond to biotic stresses [34]. Although lncRNAs may play a broadly critical role in coordinating growth and development, as well as in abiotic and biotic responses, the biological significance of lncRNA movements remains largely elusive, with only a few studies having been carried out on the transport of lncRNAs. In plants, grafting studies have identified 22 lncRNAs which move systemically into root tips and developing leaves, where they can respond to early Pi deficiency [35]. It has also been shown that lncRNAs are transferred between different types of cells through exosomes as a means of information exchange, acting as important activators or inhibitors to regulate gene expression and participating in a variety of biological processes [36,37]. Thus, these observations that lncRNAs can move long distances through phloem to sink tissues, or move in different cells, have suggested to us the bold idea that lncRNAs might have potential mobility across species through dodder bridges, which merits further exploration.

Recently, the genomes of *C. australis* R.Br. and *C. campestris* Yunck. have been sequenced and published, thus providing useful resources for the comprehensive investigation of the evolution and physiological ecology of *Cuscuta* [1,38]. Furthermore, the whole-genome sequence of crop soybean [*Glycine max* (L.) Merr. var Williams 82], one of the known hosts of dodder, has been reported [39,40]. This evidence provides support that soybean and dodder can be used as ideal candidate parasitic systems for further investigation of the ability of haustorium-mediated lncRNA transfer between two organisms. In this study, with the transcription analysis of the dodder–soybean parasitic system, we found, for the first time, that lncRNAs were translocated between the stems of the two species. Among the target genes of mobile lncRNAs, hundreds of mobile lncRNA–mRNA pairs can be co-transferred between dodder and host soybean. This inter-plant lncRNA trafficking through dodder bridge connections may provide new insights into the potential regulatory roles of lncRNAs in parasitic system interactions.

## 2. Results

### 2.1. Dodder Infestation-Induced Physiology Responses in Soybean Host

**Figure 1:**
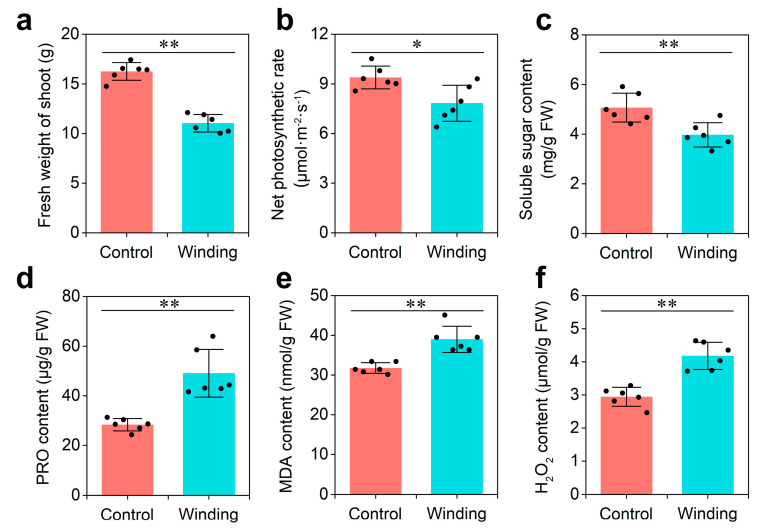
Physiological analysis of soybean in response to dodder parasitism: (**a**) Fresh weight of shoot; (**b**) net photosynthetic rate; (**c**) soluble sugar content; (**d**) proline (PRO) content; (**e**) malondialdehyde (MDA) content; and (**f**) H_2_O_2_ content. Asterisks indicate significant differences between control (soybean without dodder) and winding (soybean winded by dodder) groups, determined by Student’s *t*-test (*n* = 6; *, *p* < 0.05; **, *p* < 0.01). Error bars are ±SE.

Dodder infestation has severe effects on the growth of its host. To explore the physiological responses of hosts to dodder parasitism, two-week-old soybean seedlings were infested with dodder (winding group) or mock-treated (control group) for 3 weeks. Compared with those in the control group, the fresh weight of shoots, net photosynthetic rate, and soluble sugar content of soybean infested by the dodder decreased significantly in the winding group (Figure 1a–c). In contrast, proline (PRO), malondialdehyde (MDA), and H_2_O_2_ contents in the winding group were 75%, 22%, and 33% higher than in the control group, respectively (Figure 1d–f). These data indicate that soybean plants prime themselves to respond dramatically to the dodder parasitism at the physiological level, which provides an important stepping stone in understanding lncRNA communication at the molecular level.

### 2.2. RNA Sequencing and Identification of lncRNAs

In order to determine whether there exists cross-species lncRNA transfer in the soybean–dodder parasitization system, dodder seedlings were initially twisted and spread on soybean plants. Then, the dodder stems, interface stems where the parasite was connected to the soybean, and soybean stems were collected when the parasitic system had been established (Supplementary Figure S1). Three biological repeats were performed for each group of samples, and nine samples were sequenced on an Illumina NovaSeq platform for transcriptome analysis. A total of 121.84 Gb of clean data were ultimately generated, after the removal of poor-quality reads and adapters. The clean sequences were used to identify lncRNAs present in the analyzed tissues. To this end, cleaned paired-end reads were mapped to the soybean reference genome (Wm82.a2.v1) [40] and the *C. australis* reference genome [38]. Sequences that did not match any of the genomes due to sequencing errors were filtered out. Subsequently, reads that matched to both genomes and only matched to the native genome were considered to be from native transcripts, while reads that matched the foreign plant genome but not the native plant genome were considered to be mobile transcripts. After strict screening and mapping, the mapping rates were generally greater than 85%. These results indicated that the RNA-seq reads were highly reliable (Supplementary Table S1).

**Figure 2:**
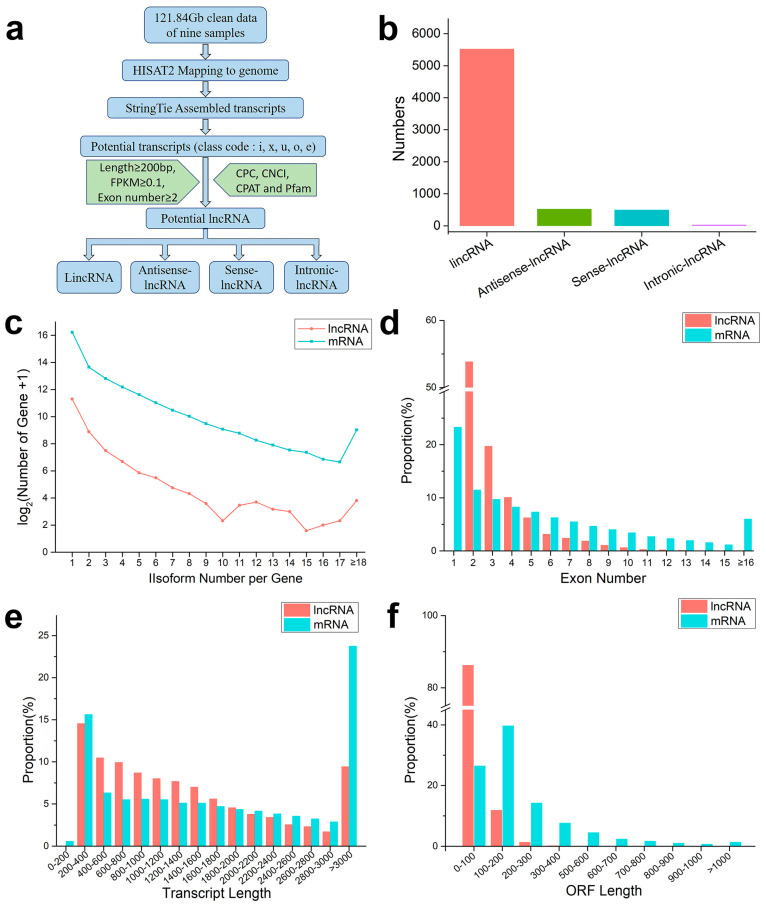
An integrative computational pipeline for the systematic identification and characterization of lncRNAs: (**a**) informatics pipeline for identification of lncRNAs; (**b**) composition of various types of lncRNAs; (**c**) number distributions of spliced lncRNAs and mRNAs; (**d**) proportion of exons per transcription for lncRNAs and mRNAs; (**e**) transcript length distributions for all lncRNAs and mRNAs; and (**f**) open reading frame (ORF) distributions for all lncRNAs and mRNAs.

According to the pipeline in Figure 2a, further analysis identified 6580 lncRNAs, including 1892 soybean lncRNAs and 4688 dodder lncRNAs. These lncRNAs were assigned to 5525 lincRNAs, 526 antisense lncRNAs, 497 sense lncRNAs, and 32 intronic lncRNAs, according to the anatomical properties of their gene loci (Figure 2b). Subsequently, the basic genomic features of lncRNAs and mRNAs were comparatively analyzed. We found that lncRNAs were expressed at similar levels in different groups and had fewer fragments per kilobase per million fragments mapped (FPKM) than protein-coding mRNAs in each group (Supplementary Figure S2). Among them, 54% of the lncRNAs were spliced (Figure 2c). The majority of lncRNAs (~55%) had two exons, and the number of lncRNAs decreased with an increase in the number of exons, while mRNAs contained more and more widely distributed exons: approximately 6% of mRNAs had more than 16 exons (Figure 2d). The average length of these lncRNAs (1458 bp) was shorter than that of protein-coding mRNAs (2133 bp); approximately 60% of the lncRNA lengths ranged from 200 to 1400 bp, while those longer than 3000 bp accounted for only 9% (Figure 2e). More than 90% of lncRNAs contained an open reading frame (ORF) of length ≤ 200 bp, while about of 34% mRNAs had ORF length ≥ 200 bp (Figure 2f). Overall, both the transcript length and ORF length of lncRNAs were shorter, compared with those of mRNAs.

### 2.3. Identification and Validation of Mobile lncRNAs

**Figure 3:**
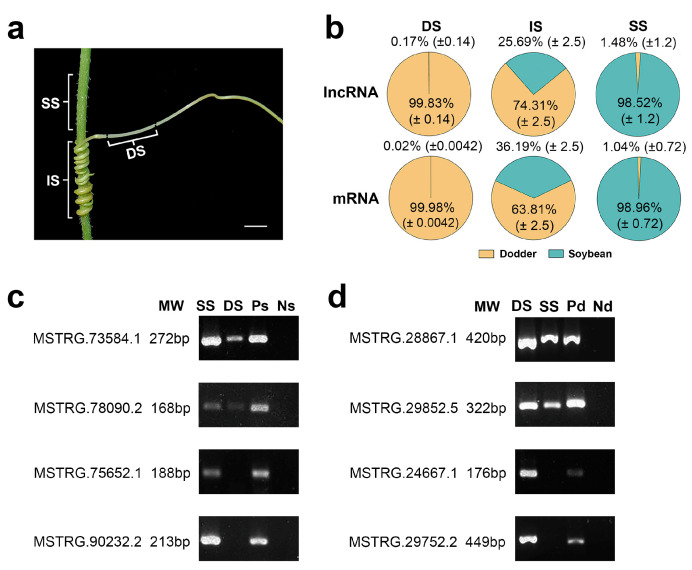
Transcript transfer in soybean–dodder parasitization systems: (**a**) Sequencing and analysis of three types of tissues, including the dodder stems (DS), interface stems (IS), and soybean stems (SS). Scale bars = 5 mm; (**b**) Pie charts illustrating the proportion of reads from foreign and native lncRNAs or mRNAs in each tissue. Calculations were based on the ratio of the reads mapped only to the foreign genome and total reads mapped to the foreign and native genomes. The values are the mean ± standard deviation of three replicates. DS, IS, and SS represent dodder stems, interface stems, and soybean stems, respectively; (**c**) RT-PCR confirmed the transfer of lncRNAs into the dodder for two soybean lncRNAs, MSTRG.73584.1 and MSTRG.78090.2; MSTRG.75652.1 and MSTRG.90232.2 were not detected in the dodder by RNA-seq. SS represents soybean stem; DS represents dodder stem; Ps represents positive control (soybean without dodder); Ns represents negative control (dodder not growing on soybean); (**d**) RT-PCR confirmed the transfer of lncRNAs into the host for two dodder lncRNAs, MSTRG.28867.1 and MSTRG.29852.5; MSTRG.24667.1 and MSTRG.29752.2 were not detected in soybean by RNA-seq. DS represents dodder stem; SS represents soybean stem; Pd represents positive control (dodder not growing on soybean); Nd represents negative control (soybean without dodder).

To explore the mobility of lncRNAs between the different plants, we used the above-developed lncRNA database to analyze the lncRNAs in three various tissues (dodder stems, soybean stems, and interface stems). In dodder stems, the proportions of the lncRNA reads from soybean averaged 0.17% of the total mapped reads across three sequencing runs, whereas soybean stems contained 1.48% dodder lncRNA reads, indicating that bidirectional movement of lncRNAs occurred between the dodder and soybean. Similarly, dodder stems contained 0.02% soybean mRNA reads, while soybean stems contained 1.04% dodder mRNA reads, suggesting that lncRNA movement is usually accompanied by mRNA trafficking (Figure 3a,b; Supplementary Table S2).

In the dodder–soybean parasitic system, the established mobile reads represent the diversity of transcripts. Subsequently, the number of mobile or non-mobile transcripts was determined, in order to compare the transferability of inter-plant lncRNAs and mRNAs. As shown in Table 1, 365 dodder lncRNAs and 8894 dodder mRNAs were detected in soybean stems, accounting for 7.8% (365/4688) and 52.4% (8894/16,977) of the total dodder lncRNAs and mRNAs, respectively. In contrast, only 14 soybean lncRNAs and 74 soybean mRNAs were identified in dodder stems, comprising 0.74% (14/1892) and 0.17% (74/42,296) of the total transcripts of soybean, respectively.

To further confirm the trafficking of inter-plant lncRNA individuals, several mobile and non-mobile lncRNA transcripts were selected and analyzed by reverse transcription-polymerase chain reaction (RT-PCR). Mobile lncRNAs MSTRG.73584.1 and MSTRG.78090.2 from soybean were detected in dodder stems at a lower level than in soybean stems; similarly, mobile lncRNAs MSTRG.28867.1 and MSTRG.29852.5 from dodder were detected in soybean stems at a lower level than in dodder stems. In contrast, non-mobile lncRNAs were detected only in soybean stems or dodder stems (Figure 3c,d). The RT-PCR results indicated that the lncRNA data obtained by RNA-seq were reliable.

**Figure 4:**
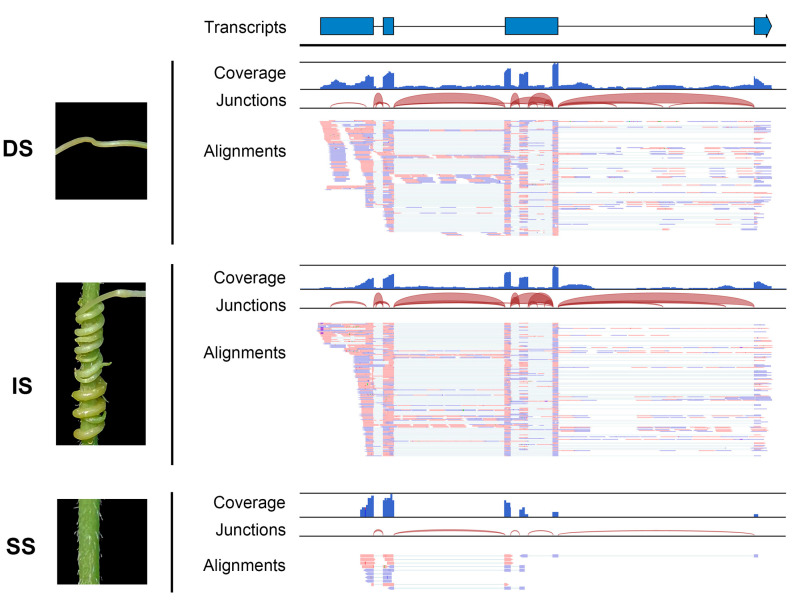
Visualization of read assemblies of the dodder lncRNA MSTRG.10219.19 in three tissues. The lncRNA model at the top indicates exons as blue bars and introns as line bridges. Each panel includes tracks for total coverage, junction coverage, and read alignments. Reads that span junctions are connected with thin lines. DS, IS, and SS represent dodder stems, interface stems, and soybean stems, respectively.

Additionally, the read coverage and alignments of RNA-seq data illustrated the form of the mobile transcripts. The read sequences and coverage of mobile lncRNA MSTRG.10219.19 from dodder stem tissue closely matched those of the interface tissue, with the exception that the mobile lncRNAs in the soybean stem tissue appeared in a fully spliced mature form; introns were only found in the libraries of dodder stems or interface tissues (Figure 4). This further confirmed the actual movement of lncRNAs between the dodder and soybean. Notably, although the output of read mapping itself produced an attractive picture of lncRNA movement, such confirmation is not practical for all mobile lncRNAs.

### 2.4. General Properties of the Mobile Transcripts

**Figure 5:**
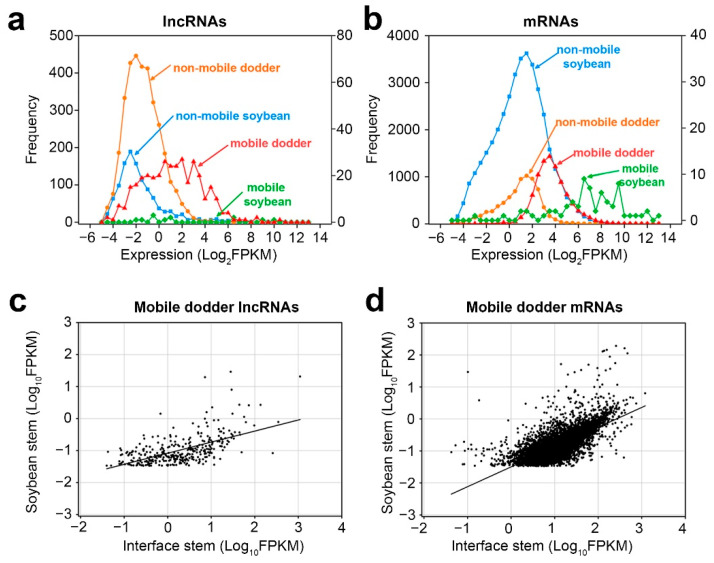
Properties of mobile and non-mobile transcripts: (**a**,**b**) distribution of lncRNAs (**a**) and mRNAs (**b**) transcript levels in interface stems related to mobility in dodder–soybean associations; (**c**,**d**) Scatter plots of lncRNAs (**c**) and mRNAs (**d**) transcript levels in the soybean stem versus those in interface stems. A total of 365 dodder lncRNAs were transferred into soybean, whereas 8894 dodder mRNAs were transferred into soybean. Lines correspond to linear regression analysis of the data.

Next, we investigated whether the inter-plant mobile transcripts possess certain properties that enable them to be transferred. First of all, by comparing the expression abundance of mobile and non-mobile transcripts in the interface stems, we found that the abundance of mobile lncRNAs was higher than that of non-mobile lncRNAs in the interface stems, and the expression patterns of mRNAs were similar to those of lncRNAs (Figure 5a,b).

Secondly, as there were only a small number of mobile soybean transcripts, correlation analysis was only performed for the transcript levels of mobile dodder lncRNAs and mRNAs in interface stems and soybean stems, respectively (Figure 5c,d). The results showed that the expression levels of mobile dodder lncRNAs or mRNAs in interface stems had a positive linear correlation with those in soybean stems. Nonetheless, the mobility pattern of lncRNAs was more dispersed, whereas the mobility pattern of mRNAs was more focused around the regression line, indicating that the dynamics of transmission of lncRNAs may differ from those of mRNAs (Figure 5c,d).

### 2.5. Functional Prediction of Mobile lncRNAs by Their Target Genes

**Figure 6:**
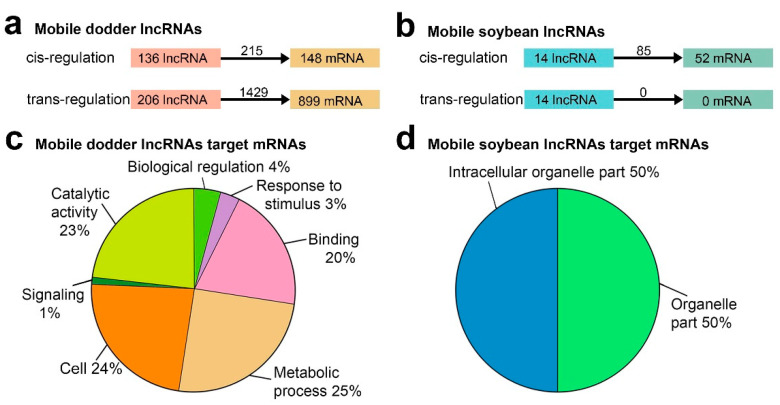
Functional analysis of mobile lncRNAs in parasitic systems: (**a**) Schematic diagram of mobile dodder lncRNAs regulating mRNAs; (**b**) Schematic diagram of mobile soybean lncRNAs regulating mRNAs. The numbers of regulatory relationship pairs are shown on the black arrows; (**c**) Pie charts showing the percentages of Gene Ontology (GO) slim terms enriched by mobile dodder lncRNAs target genes by WEGO 2.0 (*p*-value < 0.05); (**d**) Pie charts showing the percentages of GO slim terms enriched by mobile soybean lncRNAs target genes by WEGO 2.0 (*p*-value < 0.05). The full list of GO slim terms for these data is presented in Supplementary Table S4a.

To investigate the potential systemic roles of transfer lncRNAs, the target genes of transfer lncRNAs were predicted. LncRNAs spaced near protein-coding genes could participate in transcriptional regulation by binding to promoters and other *cis*-acting elements [27]. Thus, we first searched for the upstream and downstream 100 kb regions of lncRNAs and found that 136 mobile dodder lncRNAs might regulate 148 mRNAs with 215 lncRNA–mRNA pairs in *cis*, and that 14 mobile soybean lncRNAs might regulate 52 mRNAs with 85 lncRNA–mRNA pairs in *cis*, respectively (Figure 6a,b; Supplementary Table S3). Recently published data has suggested the great potential of detecting lncRNA-mediated regulation by base pair complementarity [41], which was determined to identify *trans*-acting lncRNAs. In total, 206 mobile dodder lncRNAs might regulate 899 mRNAs with 1429 lncRNA–mRNA pairs in *trans* (Figure 6a,b; Supplementary Table S3). Furthermore, the expression patterns of mobile lncRNA target genes associated with three different tissues (dodder stems, interface stems, and soybean stems) were further analyzed using the MultiExperiment Viewer 4.9 (MEV 4.9) software (Supplementary Figure S3). In addition, a total of 440 dodder target genes, including 70 (47.3%) *cis*-target genes and 370 (41.2%) *trans*-target genes, were predicted to be co-transferred with 159 mobile lncRNAs from dodder into soybean, while only four soybean *cis*-target genes were predicted to be co-transferred with 11 mobile lncRNAs from soybean into dodder (Supplementary Figure S4; Supplementary Table S3).

In order to gain insight into the function of these mobile lncRNAs, we then applied Gene Ontology (GO) enrichment to analyze their predicted target genes. A total of 12 mobile dodder and two mobile soybean GO terms were enriched by WEGO 2.0 (*p*-value < 0.05; Supplementary Table S4a). Notably, the great majority of the target genes of mobile dodder lncRNAs were enriched in “metabolic process”, “catalytic activity”, “signaling”, and “response to stimulus” categories, whereas the genes corresponding to mRNAs targeted by mobile soybean lncRNAs were only enriched in organelle-related categories, including “intracellular organelle part” and “organelle part” (Figure 6c,d). In addition, the GO enrichment analysis of these target mRNAs was also performed using the agriGO 2.0 website, the results of which were similar to those found in WEGO enrichment analysis (Supplementary Figure S5).

We also applied GO enrichment to assess the functional significance of mobile mRNAs using the WEGO 2.0 and agriGO 2.0 websites (Supplementary Table S4b). Similarly, most of the mobile dodder mRNAs were enriched in “metabolic process”, “catalytic activity”, “binding”, “biological regulation”, “response to stimulus”, and “signaling” categories (Supplementary Figure S6a; Supplementary Figure S7), while the mobile soybean mRNAs were similar to the corresponding lncRNAs, with some of them being enriched in “organelle” and “binding” categories (Supplementary Figure S6b; Supplementary Figure S7).

### 2.6. Identification of Transcription Factors of the Mobile Transcripts

**Figure 7:**
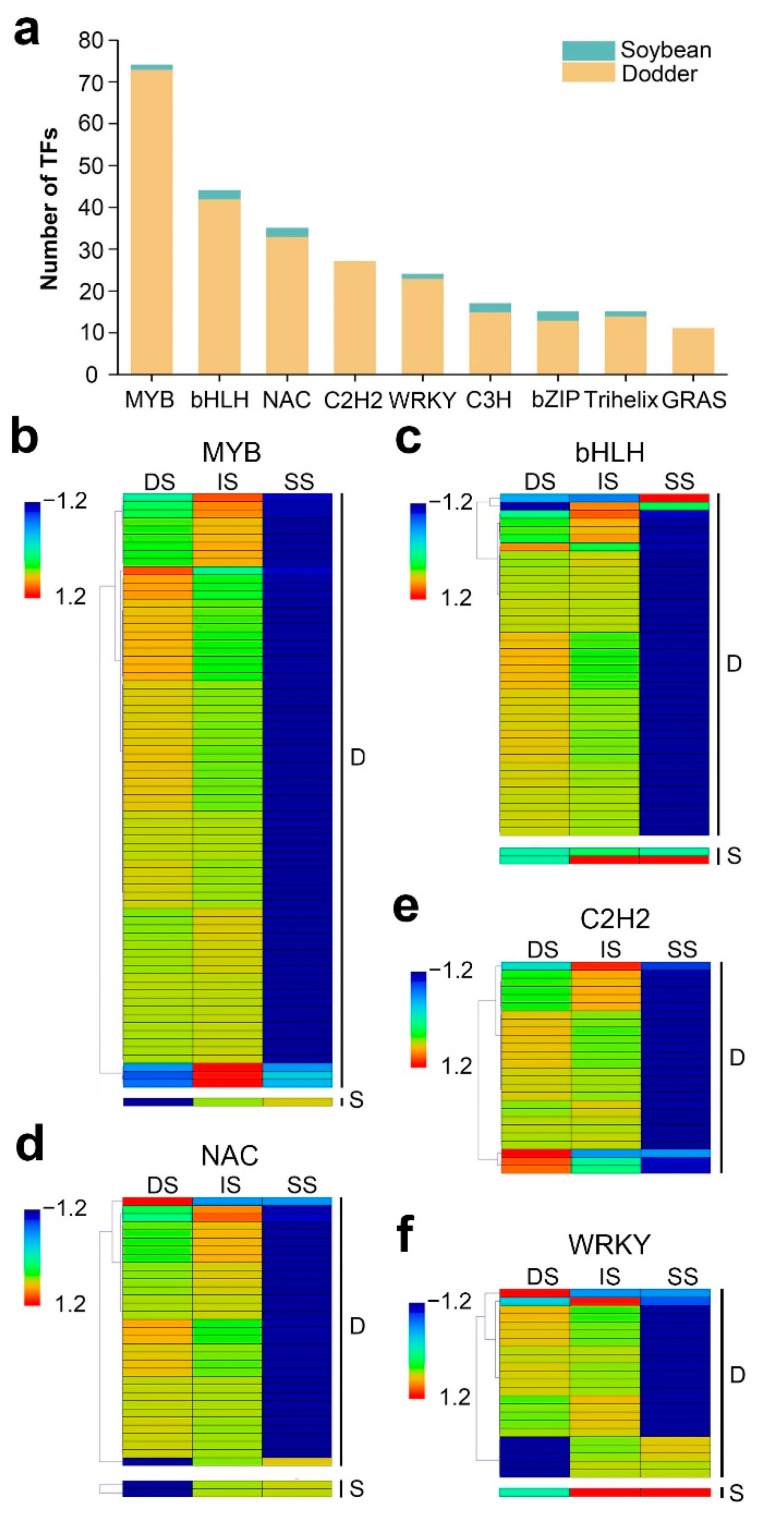
Distribution and expression patterns of transcription factors: (**a**) Details of the number for TFs identified from mobile lncRNAs target genes of dodder and soybean. Data are sorted by number of lncRNAs. Only categories with more than 10 mobile transcripts identified as transcription factors are shown; (**b**–**f**) Heatmap of the expression patterns of the first five most numbers of TFs in the tissues of dodder stems (DS), interface stems (IS), and soybean stems (SS), including MYB (**b**), bHLH (**c**), NAC (**d**), C2H2 (**e**), and WRKY (**f**). ‘D’ represents mobile dodder lncRNAs target genes. ‘S’ represents mobile soybean lncRNAs target genes. The gene expression is based on the z-scores of log_2_(FPKM) value. The blue and red colors indicate low and high expression levels, respectively.

Transcription factors (TFs) are regulatory proteins that can activate or inhibit target genes and which participate in biotic or abiotic stress responses [42,43,44,45]. To further reveal the potential regulation functions of lncRNAs, we screened the TFs corresponding to their target mRNAs. In total, 201 mobile lncRNAs resulted in the identification of 635 targeted TFs, belonging to 49 TF families (Supplementary Table S5a). In the dodder–soybean parasitic system, the MYB family was the largest gene family identified (74 in total), corresponding to 34 mobile lncRNAs, followed by the bHLH, NAC, C2H2, and WRKY families and presenting a high number of mobile transcripts (Figure 7a). The dynamic changes in the expression levels of these TFs in the three different tissues are shown in Figure 7b–f. In addition, when we screened the TFs for the mobile mRNAs, 54 TF families, including 297 mobile mRNAs, were shown to be transferred from dodder to soybean, while no TFs were predicted to be transferred from soybean to dodder (Supplementary Table S5b). We found a total of 30 TF families that were common to mobile lncRNA-targeted mRNAs and mobile mRNAs (Supplementary Table S5).

### 2.7. Potential lncRNA–mRNA/TF Network in Parasitic System

**Figure 8:**
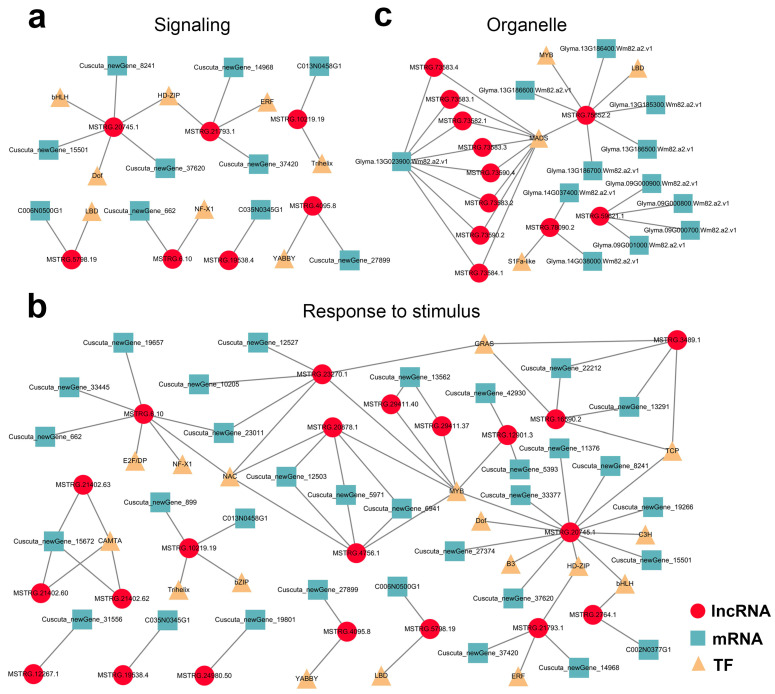
Potential lncRNA–mRNA/TF network in parasitic system visualized using Cytoscape 3.7.2: (**a**,**b**) Predicted network of mobile dodder lncRNAs and their targeted mRNAs/TFs enriched in “signaling” term (**a**) or “respond to stimulus” term (**b**); (**c**) Predicted network of mobile soybean lncRNAs and their targeted mRNAs/TFs enriched in “organelle part” term. Red circles represent lncRNAs, blue squares represent mRNAs, and yellow triangles represent TFs.

Finally, mobile lncRNA-associated networks were constructed based on all the target genes, revealing the intricacy of their relationships (Supplementary Figure S8). Among them, the detailed interactions of the mobile dodder lncRNAs enriched in “signaling” and “respond to stimulus” terms (Figure 8a,b), and the interactions of the mobile soybean lncRNAs enriched in organelle-related terms were visualized (Figure 8c). In these networks, lncRNA target prediction revealed the presence of 5 and 24 potential lncRNA–mRNA target pairs that co-transferred from dodder to soybean in “signaling” and “respond to stimulus” terms, respectively. In contrast, there were 19 potential lncRNA–mRNA/IF target pairs enriched in organelle-related terms, but no lncRNA–mRNA/TF target pairs appeared to be co-transferred.

## 3. Discussion

Taking advantage of the significant phylogenetic distances between dodder and its wide range of host species, dodder–host systems can be used as ideal parasitic systems for the identification of mobile molecules—including DNA, RNA and proteins [7,11,13,46]—and for the study of systemic signaling [8,9,10,12]. In this paper, we provide evidence that lncRNAs can also be transferred between species, moving in a bidirectional manner through dodder bridges.

Genome-wide identification studies of lncRNAs have been performed in numerous plants, including model plants, trees, and crops [47,48,49]. As essential regulators of development and stress responses, plant lncRNAs have also been shown to regulate biotic and abiotic stresses in genetic studies [30,31,32,33,50]. In this study, we identified lncRNAs from the stems of different species, including dodder stems, interface stems, and soybean stems, using the Illumina NovaSeq platform. As a result, a total of 6580 lncRNAs were identified in the dodder–soybean parasitic system. It was shown that most of these lncRNAs were lincRNAs, which might be due to genes only occupying a small part of the chromosome sequence [51]. The expression levels of lncRNAs were lower than mRNAs encoding proteins, consistent with previous studies on *Cleistogenes songorica* (Roshev.) Ohwi and *Melilotus albus* Medik. [27,52]. Moreover, the number of exons (mostly two exons), transcript length, and ORF length of lncRNAs are generally smaller than mRNAs, which may be the reason for the differences in their function and evolution [53,54,55].

Previously, mRNAs have been reported to transport between different plants through dodder bridges [11,56]. For instance, RNA-seq analysis indicated that more than 8500 and 9500 unigenes originating from dodder (*C. pentagona* Engelm.) and *Arabidopsis* were detected in *Arabidopsis* and dodder, respectively; in contrast, 347 and 288 mobile unigenes were shown to be transferred in the tomato–dodder system [11]. Liu et al. (2020) have also confirmed 172 and 1416 mobile mRNAs in dodder (*C. australis*) and *Arabidopsis*, as well as 64 and 708 mobile mRNAs in dodder and soybean [7]. In our RNA-seq analysis, both lncRNAs (365 for dodder and 14 for soybean) and mRNAs (8894 for dodder and 74 for soybean) were shown to move between dodder and soybean, strongly suggesting that dodder is capable of transmitting lncRNAs between the two species, as with the mRNAs. It is worth noting that the proportion of host/dodder lncRNAs (3.84%), as well as mRNAs (0.83%), is much lower than that previous reported (more than 82.99%) [7,11], indicating that the number of mobile mRNAs varies significantly in different parasitic systems, or even in different studies on the same parasitic system. This may be because plants at different growth stages and with different distances between sampled segments and interface regions were used in these studies. Furthermore, lncRNAs and mRNAs with relatively high abundance have a greater likelihood of moving into the other species than those with relatively low abundance in the soybean–dodder system (Figure 5a,b). This result is in line with previous findings that the abundance of mRNAs or proteins is likely a factor that influences their mobility [7,11], strongly supporting the selective mobility of RNAs and proteins in dodder bridge connections. Interestingly, we also found that mobile RNAs with high abundance in dodder appear to be more expressed when they move into the host (Figure 5c,d). These findings suggest that abundance is a major driving force for the inter-plant mobility of macromolecules.

Unlike mRNA sequences, which can provide information to predict mRNA functions, the sequence motifs of lncRNAs do not support functional prediction [53,57]. Generally, lncRNAs can act in *cis* or *trans* roles through their sequence complementarity to RNA or DNA—either as scaffolds or decoys—to regulate gene translation and expression [58]. In this study, there were 150 mobile lncRNAs *cis*-targeting 200 genes within 100 kb of their upstream and downstream, and 206 mobile lncRNAs *trans*-targeting 899 genes, based on the complementary base-pairing (Figure 6), suggesting that they may participate in interspecific communication by both *cis* and *trans* regular manners. Notably, in plants, lncRNAs can serve as small RNA precursors in RNA interference [59], interact with the chromatin remodeling complex to change the chromatin structure [60,61], act as an enhancer of translation [62], and be involved in RNA processing [63]. LncRNAs can also bind to proteins and assemble as a complex platform, or regulate protein–protein interactions [64,65]. Thus, it should not be ignored that the mobile lncRNAs identified in our study may have some additional regular functions, such as RNA interference and binding proteins, which could also regulate the biological processes in new species; this deserves exploration in future research.

Although whether these long-distance mobile lncRNAs have activities and functions in manipulating the host physiology remains to be determined, the following two lines of indirect evidence strongly support the notion that some of the mobile miRNAs and proteins still have biological functions after long-distance translocation: (1) Using transgenic hosts expressing eGFP-GUS, GUS, LUC, PAT, and EPSPS, it has been demonstrated that these proteins retained their activity after inter-plant movement [7]; and (2) several *Arabidopsis* mRNAs are targeted by 22-nucleotide dodder miRNAs during parasitism, resulting in mRNA cleavage, secondary siRNA production, and decreased mRNA accumulation [13]. Given that dodder has a very wide host range (across many plant families), it is logical to speculate that lncRNAs with the same functions can be exchanged or transferred through the haustorial connections between dodder and hosts. In this study, GO analysis on the *cis*- and *trans*-target genes of mobile lncRNAs revealed that mobile dodder lncRNA target genes were mainly enriched in “catalytic activity”, “response to stimulus”, and “signaling” terms, whereas mobile soybean lncRNA target genes were enriched in organelle-related categories (Figure 6). Many plant pathogens secrete effector proteins into plant cells to suppress the host plant’s defenses [66]. In contrast, studies on the secretomes of soybean have also indicated that the host mobile proteins may function in the recipient dodder by modulating their physiology, such as altering the growth and development of subsequent generations [7]. Together with our results, these observations thus favor a scenario in which mobile transcripts transferred from the dodder into the host might participate in signaling and nutrient redistribution, while the host mobile lncRNAs may provide feedback which, in turn, influences the growth and development of the dodder (e.g., haustorium establishment) [7].

TFs are vital regulators that can bond with corresponding *cis*-acting elements to modulate their target gene functions, including responses to biotic stresses and environmental factors [67]. Recent studies have provided a comprehensive update on wheat TFs involved in defense responses against pathogen infection [68]. In this paper, 49 TF families corresponding to 635 targeted TFs were identified. Among them, the MYB, bHLH, NAC, C2H2, and WRKY families, which have been revealed to respond to biotic stress and growth in plants [43,44,45], accounted for a large proportion of these TFs. These results are similar to those of previous proteome analyses on the transferability of MYB, bHLH, C_2_H_2_, bZIP, and ARF TFs between host and dodder plants [7], suggesting their high biological importance in forming a stable parasitic system.

**Figure 9:**
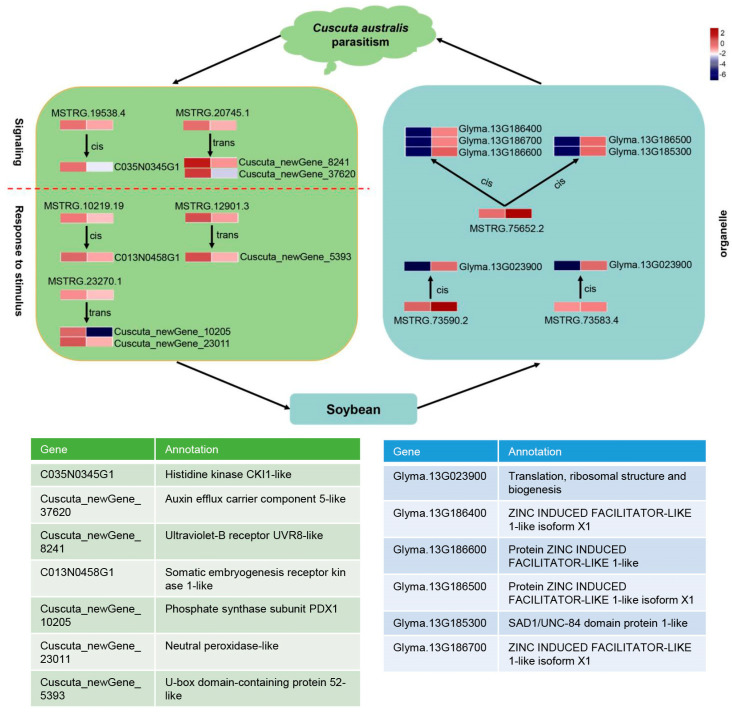
The inferred lncRNA–mRNA interaction pathway of host soybean in response to dodder parasitism. Heatmap of the expression level of the mobile lncRNAs and targeted mRNAs in the tissues of dodder and soybean stems. The gene expression is based on the z-scores of log_10_(FPKM) value. Below are the gene annotations of important target genes enriched in three terms, including “signaling”, “response to stimulus”, and “organelle part”. The green and blue colors indicate dodder and soybean transcripts, respectively.

The inferred lncRNAs–mRNAs/TFs network could be a potential mechanism regulating parasite–host plant interactions. With such networks, researchers are not only able to evaluate the functions of lncRNA by means of well-studied protein-coding mRNA, but can also deduce the lncRNAs related to mRNAs of interest. Indeed, we found that dodder lncRNAs related to “signaling” and “respond to stimulus” were transferred to soybeans, and that some of these mobile lncRNAs and targeted mRNAs were co-transported to soybeans, such as the pairs of MSTRG.20745.1 and Cuscuta_newGene_8241 (HD-ZIP) as well as MSTRG.10219.19 and C013N0458G1 (Trihelix) (Figure 8 and Figure 9). Consistently, at the physiological level, we found that dodder parasitism resulted in marked oxidative damage in soybeans, meaning that ROS levels (mainly including H_2_O_2_) accumulated markedly, and that lipid peroxidation (MDA content) was significantly aggravated in soybean plants. To alleviate oxidative damage, the soybean antioxidant defense system was significantly activated using osmotic adjustment substance (PRO) to scavenge the over-accumulated cellular ROS to relatively low levels. Meanwhile, several soybean organelle-related lncRNAs were transferred to dodder, such as the pairs of MSTRG.75652.2 and Glyma.13G186700.Wm82.a2.v1 (LBD TF) as well as MSTRG.73584.1 and Glyma.13G023900.Wm82.a2.v1 (MADS TF) [69,70] (Figure 8 and Figure 9). Whether these co-transferred lncRNAs–mRNAs/TFs have functions in the recipient plants remains to be elucidated. Further studies can select several of the lncRNAs–mRNAs/TFs modules mentioned above but not limit them for functional analysis by reverse genetics, using existing mutant populations or through genome editing.

**Figure 10:**
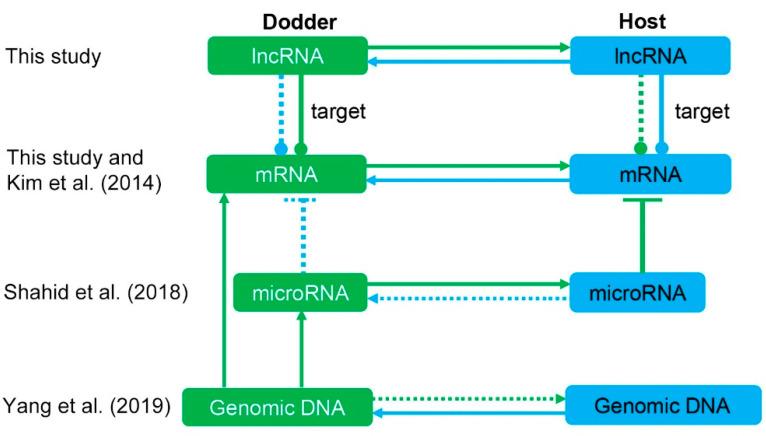
Mobile molecules in light of interaction with RNAs and DNA. Green represents parasite, blue represents host. Solid lines represent direct evidence, dashed lines represent potential pathways that currently lack evidence in this system. Kim et al. (2014), Shahid et al. (2018), and Yang et al. (2019) represent supporting references: [11], [13], and [46], respectively. Bidirectional transfer of lncRNAs was observed by RNA sequencing in this study, but the cross-species targeting of mobile lncRNAs to foreign genes requires further experimental validation.

Taken together, this study demonstrates that lncRNAs can be translocated bidirectionally between dodder and its host, and some mobile lncRNAs and their predicted target mRNAs can co-transfer during parasitism (Figure 10; Figure S4). Although the functional effects of these movements—especially lncRNA–mRNA interaction modules—need to be further explored, the large-scale transfer of lncRNAs between parasite and host affects their exchange of various substances and, therefore, likely play a key role in the establishment and maintenance of parasitism. Dodder-mediated cross-species mobility of lncRNAs will provide a critical database for further analysis of the parasitic systemic function of plant lncRNAs.

## 4. Materials and Methods

### 4.1. Plant Material and Sampling

Soybean seeds of Williams 82 and dodder seeds of *C. australis* were kindly provided by Professor Bin Liu (Institute of Crop Sciences, Chinese Academy of Agricultural Sciences, Beijing, China) and Professor Jianqiang Wu (Kunming Institute of Botany, Chinese Academy of Sciences, Kunming, China), respectively. Soybean plants (*G. max* var Williams 82) were grown in a greenhouse under a 12 h photoperiod (light intensity ~800 μmol/m^2^/s) at 26 ± 2 °C and 60 ± 5% relative humidity. The seeds of *C. australis* were submerged in sulfuric acid for 30 min, then rinsed with water 10 times. The germinated *Cuscuta* seedlings were twisted and spread on 2-week-old soybean plants. Three weeks after the initiation of infestation (Supplementary Figure S1), three distinct regions were collected for strand-specific RNA sequencing. As shown in Figure 3a, the three tissues included interface regions where the haustoria were bound tightly to host tissues, 2 cm *Cuscuta* stem segments 1 cm away from the attachment region, and 1 cm soybean segments 1 cm above the attachment region. Three biological replicates were sampled for each tissue; each sample consisted of a pool of 3–5 stem segments. All samples were immediately frozen in liquid nitrogen and stored at −80 °C.

The photosynthetic index was measured using a LI-6400 portable photosynthesis system (LI-COR, Lincoln, NE, USA) from 9:00 to 11:00 a.m. Physiological indicators, such as soluble sugar, PRO, MDA, and H_2_O_2_, were determined using corresponding reagent kits (KT-1-Y, PRO-1-Y, MDA-1-Y, H_2_O_2_-1-Y; Cominbio, Suzhou, Jiangsu, China; http://www.cominbio.com/, accessed on 13 March 2020).

### 4.2. cDNA Library Construction and Sequencing

Total RNA from nine independent samples (three biological replicates of three tissue groups) was isolated separately using TRIzol reagent (Invitrogen, Waltham, MA, USA), according to the manufacturer’s instructions. A Ribo-Zero™ kit (Epicentre, Madison, WI, USA) was used for rRNA removal from the total RNA sample, when the concentration, integrity, and purity of the RNA samples were all qualified. Afterwards, the total RNA from all samples was used to construct cDNA libraries using an Illumina NEBNext^®^ Ultra^TM^ Directional RNA Library Prep Kit (NEB, Ipswich, MA, USA). A total of nine libraries were sequenced on an Illumina NovaSeq platform with 2 × 150 bp paired-end reads [71]. Clean data were obtained by removing reads containing contaminating read adapters, poly-N, low-quality, and poor-quality reads from the raw data.

All libraries were assumed to contain a mixture of soybean and dodder sequences. To confirm RNA transfer from soybean to dodder, HISAT2 (http://ccb.jhu.edu/software/hisat2/index.shtml, accessed on 7 February 2020) was used to map cleaned reads from the dodder samples to the *C. australis* genome, and the unmapped reads were matched against the soybean reference genome (Wm82.a2.v1) [40]; if it did not match either genome (for example, due to sequencing errors), it was filtered out. The resulting soybean sequences were identified as mobile RNAs from soybean to dodder. Mobile RNAs of soybean samples were identified in a similar manner. The mapped reads from each library were assembled using the StringTie software (http://ccb.jhu.edu/software/stringtie/, accessed on 13 February 2020) and the FPKMs of both lncRNAs and mRNAs were calculated [72]. 

LncRNAs were identified based on their characteristics, according to the pipeline (Figure 2a) as follows [73,74]: (1) the class_code of transcripts was selected as “i”, “x”, “u”, “o” or “e”; (2) transcripts with length ≥200 nt and exon count ≥2 were selected; (3) transcripts with FPKM ≥0.1 were selected; (4) transcripts that passed the protein-coding score test with Coding Potential Calculator (CPC), Coding-Non-Coding Index (CNCI), and the Coding Potential Assessment Tool (CPAT) were removed; and (5) the remaining transcripts that contained protein-coding domains were removed by alignment with the Pfam databases [75,76,77,78]. Various types of lncRNAs, including lincRNAs, antisense lncRNAs, sense lncRNAs, and intronic lncRNAs, were identified using cuffcompare [79]. All sequencing reads generated from the Illumina NovaSeq platform are available in NCBI SRA: SRR15100082-90 (https://www.ncbi.nlm.nih.gov/sra, accessed on 14 July 2021).

### 4.3. Analysis of Mobile lncRNAs and mRNAs

The *cis* and *trans* target genes of mobile lncRNAs were predicted, in order to analyze their functions. Protein-coding genes spaced less than 100 kb upstream and downstream of lncRNAs were identified to predict putative target neighboring genes of *cis-acting* lncRNAs, and the LncTar software was used to analyze the complementary base pairing between lncRNAs and mRNAs [80].

Venn diagram analyses were performed through an online platform (http://bioinfogp.cnb.csic.es/tools/venny/, accessed on 23 May 2021). The cluster analysis and expression pattern were carried out using the MEV 4.9 software through the hierarchical clustering and the K-means clustering method. GO enrichment analysis of mobile transcripts was performed using WEGO 2.0 (https://wego.genomics.cn, accessed on 1 June 2021) and agriGO 2.0 (http://systemsbiology.cau.edu.cn/agriGOv2, accessed on 1 June 2021). TFs were predicted and classified into various families using the BMK Cloud Server platform (http://www.biocloud.net/, accessed on 7 June 2021). Finally, the regulatory networks were constructed using the Cytoscape 3.7.2 software between lncRNA–mRNA/TF [81].

### 4.4. RT-PCR Confirmation

The total RNA of all samples used for the transcriptome analysis was also used to generate cDNA for RT-PCR validation. Gene-specific primers for RT-PCR were designed using the DNAMAN software (Lynnon BioSoft, Canada, accessed on 3 July 2021), and are given in Supplementary Table S6. Three technical replicates were assayed for each sample.

## 5. Conclusions

In this study, we showed, for the first time, that lncRNAs can be translocated within the dodder–soybean parasitic system through high-throughput sequencing. A total of 6580 lncRNAs were identified, among which 365 dodder and 14 soybean lncRNAs were found in soybean and dodder stems, respectively. It was shown that these lncRNAs are selectively mobile, preferring to move when more abundant. We also predicted that 255 mobile dodder lncRNAs might regulate 1045 mRNAs, and 14 mobile soybean lncRNAs might regulate 52 mRNAs. GO enrichment showed that the mobile dodder lncRNA target genes were mainly enriched in “catalytic activity”, “response to stimulus”, and “signaling” terms, while mobile soybean lncRNA target genes were enriched in organelle-related terms. Furthermore, the inferred lncRNAs-mRNAs/TFs network may provide a potential mechanism for further analysis of the systemic function of mobile lncRNAs. Our findings not only provide new insight into the mechanism of the dodder–host interaction, but also add another means of interspecific communication to the previously identified transfer of mRNAs, microRNAs, proteins, and systemic signals.

## Figures and Tables

**Table 1:** Numbers of lncRNAs and mRNAs transferred in the soybean–dodder system.

Mobility Category	Soybean lncRNAs	Dodder lncRNAs	Soybean mRNAs	Dodder mRNAs
Total mobile	14	365	74	8894
Nonmobile	1878	4323	42,222	8083
Total	1892	4688	42,296	16,977

## Data Availability

All sequencing reads generated from the Illumina NovaSeq platform are available in NCBI SRA: SRR15100082-90 (https://www.ncbi.nlm.nih.gov/sra, accessed on 14 July 2021). Other data sets supporting the conclusions of this article are included within the article and its additional files.

## Supplementary Materials

The following are available online at https://www.mdpi.com/article/10.3390/ijms23010561/s1.
