# Supplementary material for: Bidirectional lncRNA Transfer between Cuscuta Parasites and Their Host Plant
Source: Int J Mol Sci. 2022 Jan 5;23(1):561. doi: 10.3390/ijms23010561 (PMC8745499; doi:10.3390/ijms23010561)
Supplement: Supplementary file 1 [file ijms-23-00561-s001.zip › ijms-1487347-supplementary/Supplementary Figures S1-S8.pdf]

Bidirectional lncRNA transfer between *Cuscuta* parasites and their host plant

Yuguo Wu, Dong Luo, Longfa Fang, Qiang Zhou, Wenxian Liu and Zhipeng Liu*

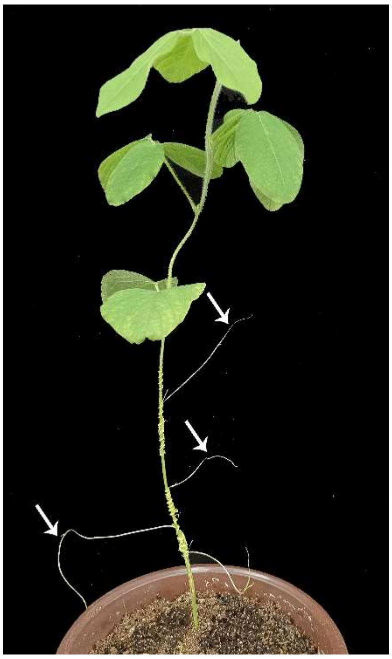

Figure S1. Overview of parasitic dodder attached to soybean plant. Arrows indicate stems of dodder.

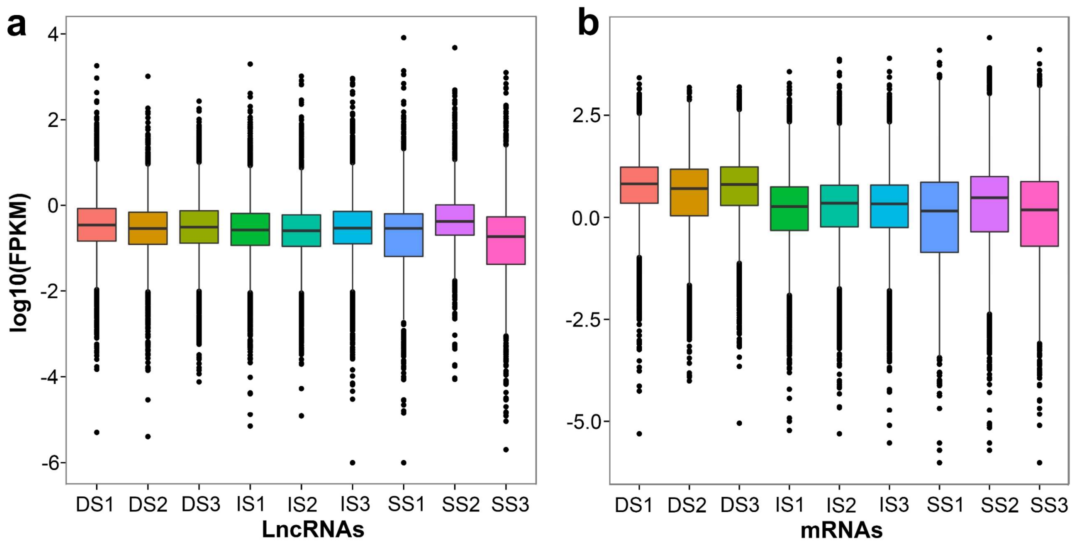

Figure S2. Box plot of the expression levels of lncRNAs (**a**) and mRNAs (**b**) in three different tissues of the dodder–soybean parasitic system. DS: Dodder stems; SS: Soybean stems; IS: Interface stems.

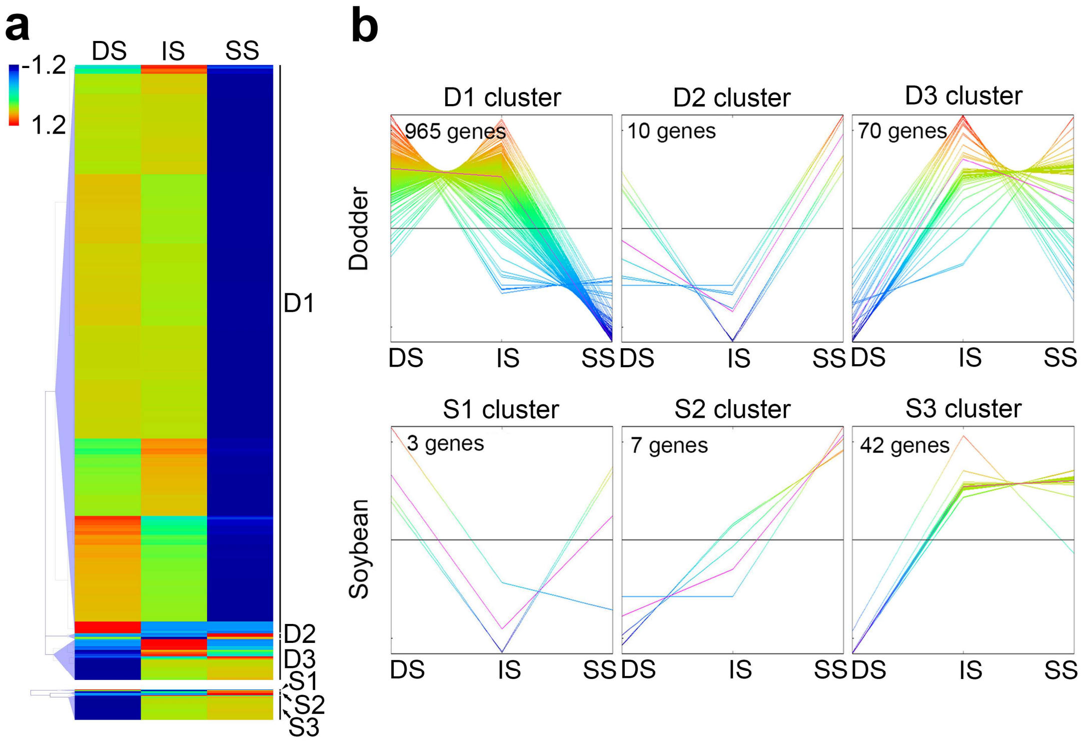

Figure S3. An overview of the dynamic changes in the expression level of mobile lncRNAs target genes associated with three different tissues, including dodder stems (DS), interface stems (IS), and soybean stems (SS): (**a**) Heatmap showing mobile lncRNAs target genes using the MEV 4.9 software with the hierarchical clustering method. Six clusters are shown, of which D1–D3 represent clusters 1–3 of dodder and S1–S3 represent clusters 1–3 of soybean; and (**b**) dynamic expression of the mobile lncRNAs target genes in each of the six clusters was analyzed by the MEV4.9 software with the K-means clustering method. The gene expression is based on the z-scores of $\log_2$(FPKM) value. The blue and red colors indicate low and high expression levels, respectively.

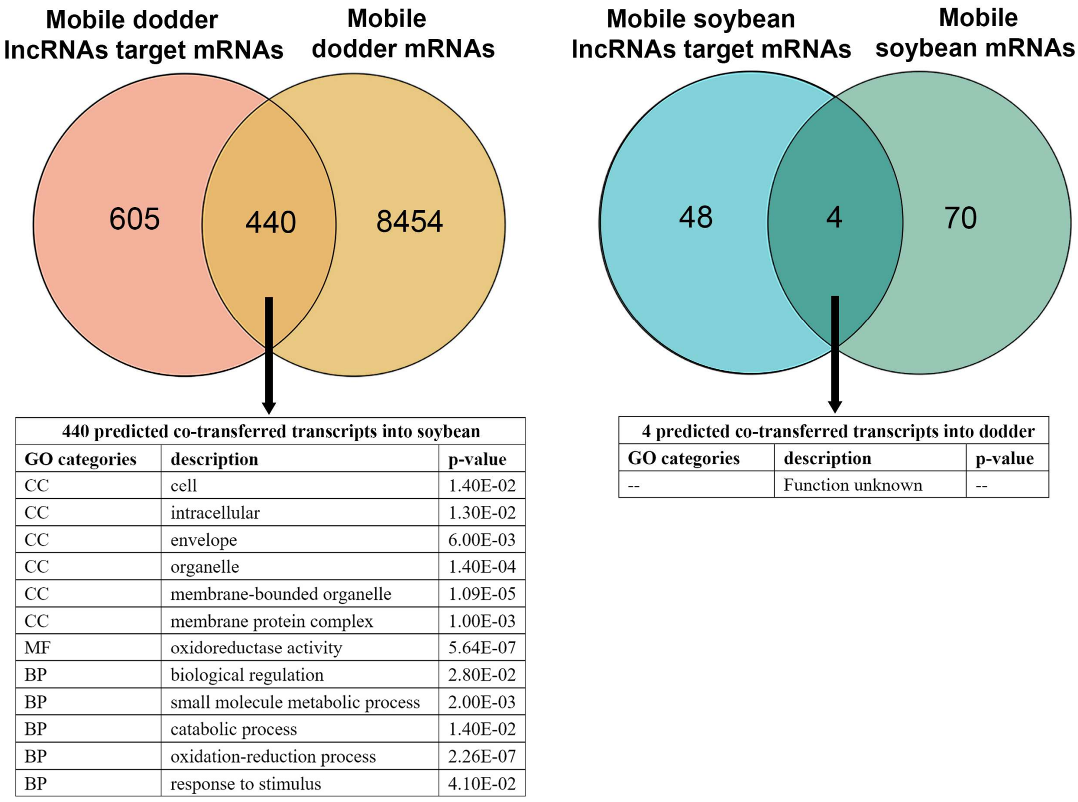

440 predicted co-transferred transcripts into soybean		
**GO categories**	**description**	**p-value**
CC	cell	1.40E-02
CC	intracellular	1.30E-02
CC	envelope	6.00E-03
CC	organelle	1.40E-04
CC	membrane-bounded organelle	1.09E-05
CC	membrane protein complex	1.00E-03
MF	oxidoreductase activity	5.64E-07
BP	biological regulation	2.80E-02
BP	small molecule metabolic process	2.00E-03
BP	catabolic process	1.40E-02
BP	oxidation-reduction process	2.26E-07
BP	response to stimulus	4.10E-02

4 predicted co-transferred transcripts into dodder		
**GO categories**	**description**	**p-value**
--	Function unknown	--

Figure S4. Functional analysis of co-transferred transcripts (the target genes predicted by mobile lncRNAs overlapped with mobile mRNAs) in parasitic system. Venn diagrams indicate the overlapping and unique mobile dodder or soybean lncRNAs target genes and mobile mRNAs. Below are the GO terms enriched from co-transferred dodder transcripts by WEGO 2.0 (*P*-value < 0.05). Four hundred and forty mobile dodder target genes were mainly associated with cell, envelope, membrane protein complex, biological regulation, catabolic process, oxidation-reduction process, response to stimulus, and oxidoreductase activity. In contrast, the four mobile soybean target genes were not enriched in known GO categories.

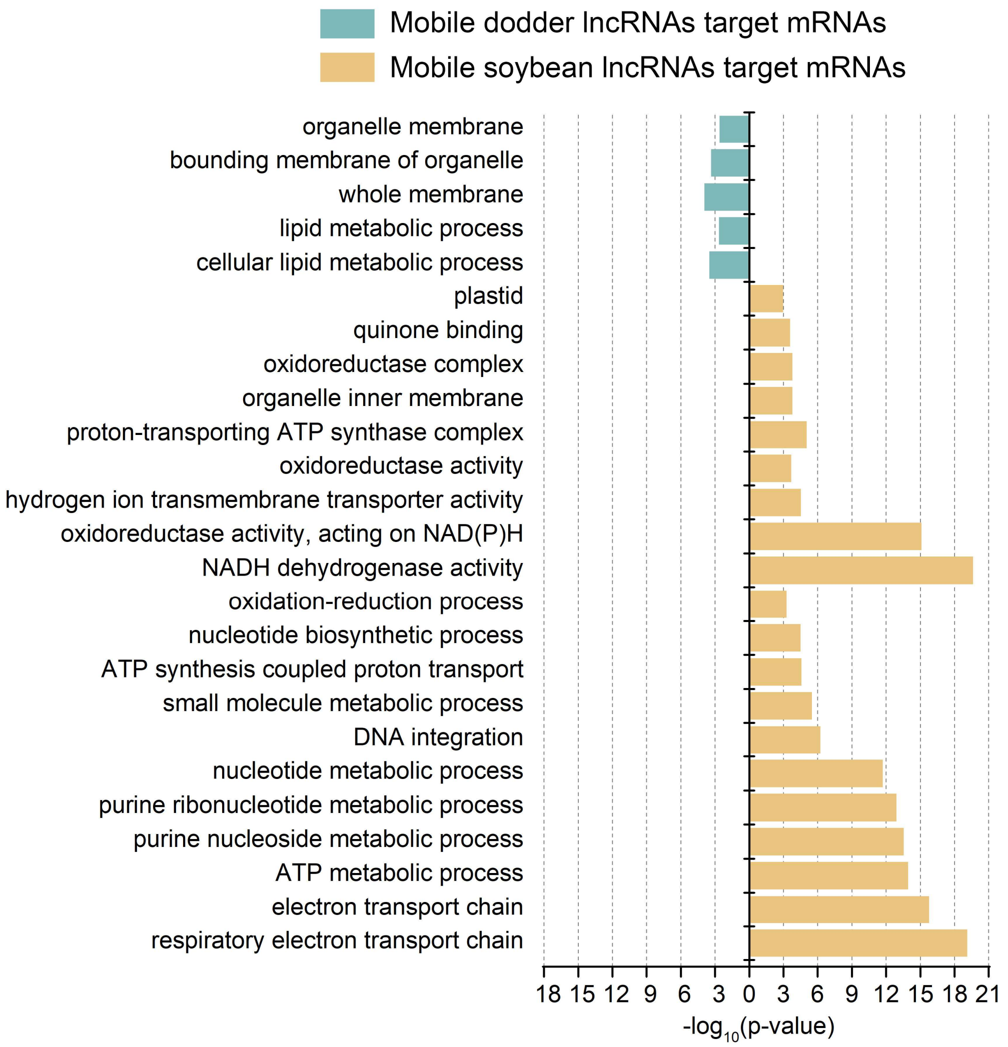

Figure S5. Gene Ontology enrichment of mobile lncRNA target genes in the soybean–dodder system using the agriGO 2.0 website. The names of the GO categories are listed along the y-axis. The degree of GO enrichment is represented by the -log$_{10}$(p-value).

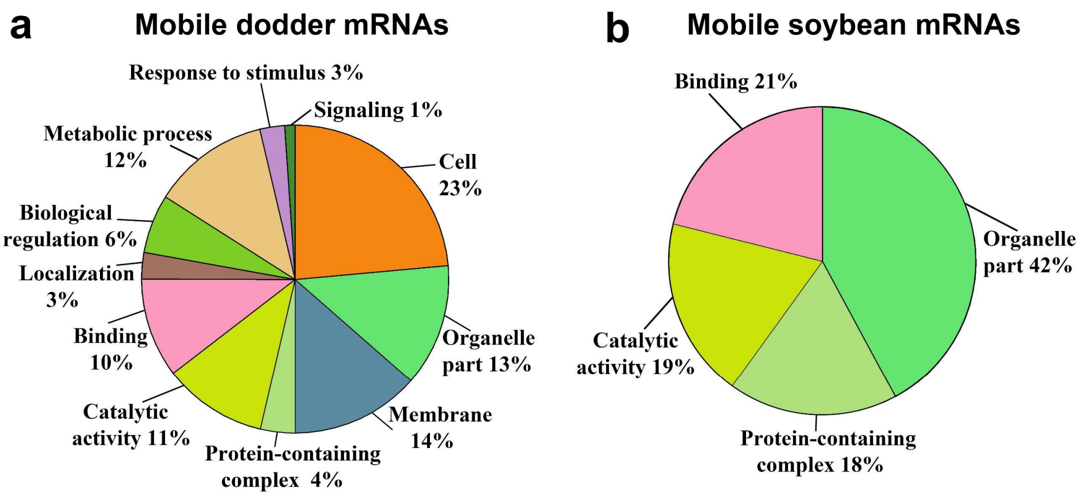

Figure S6. Pie charts showing the percentage of GO slim terms enriched by mobile dodder (**a**) or soybean (**b**) mRNAs by WEGO 2.0 (*P*-value < 0.05). The full list of GO slim terms for these data is presented in Table S4b.

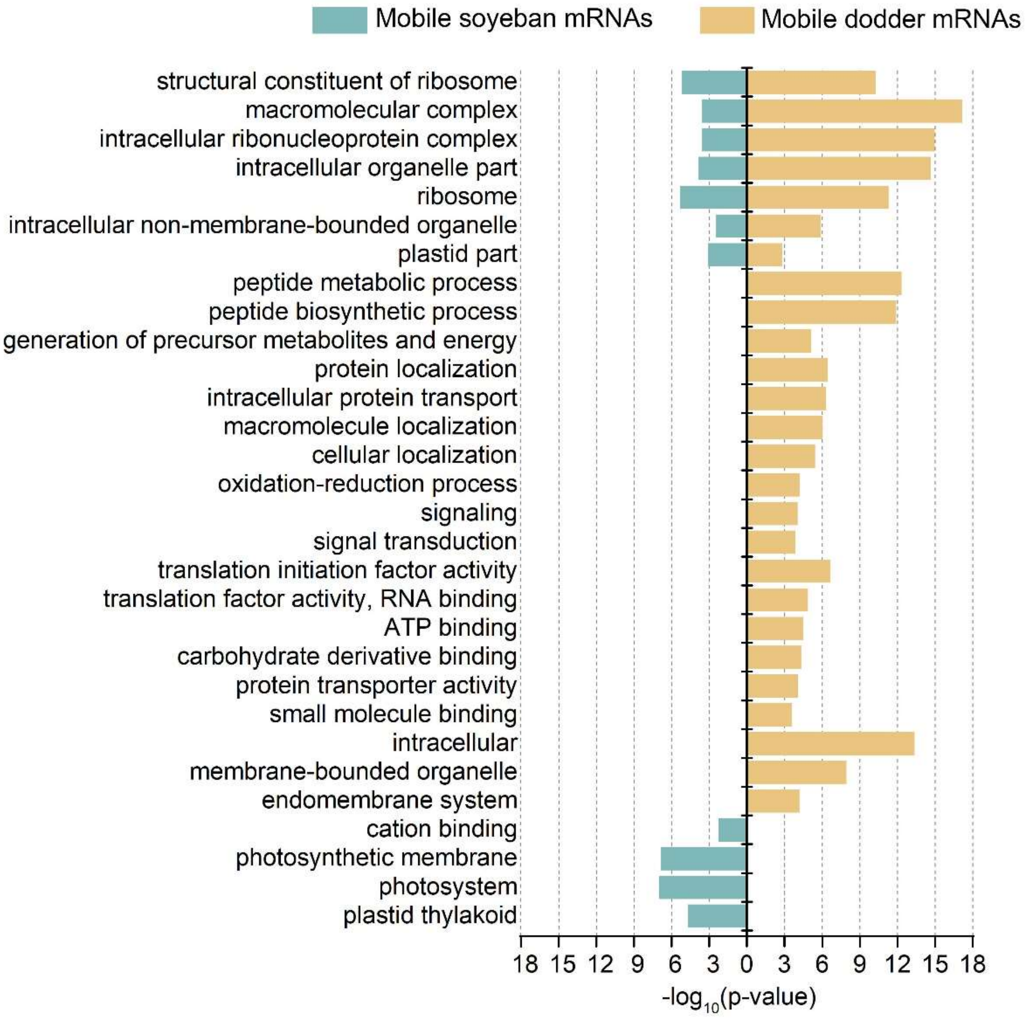

Figure S7. Gene Ontology enrichment of mobile mRNA in the soybean–dodder system using the agriGO 2.0 website. The names of the GO categories are listed along the y-axis. The degree of GO enrichment is represented by the $-\log_{10}(p\text{-value})$.

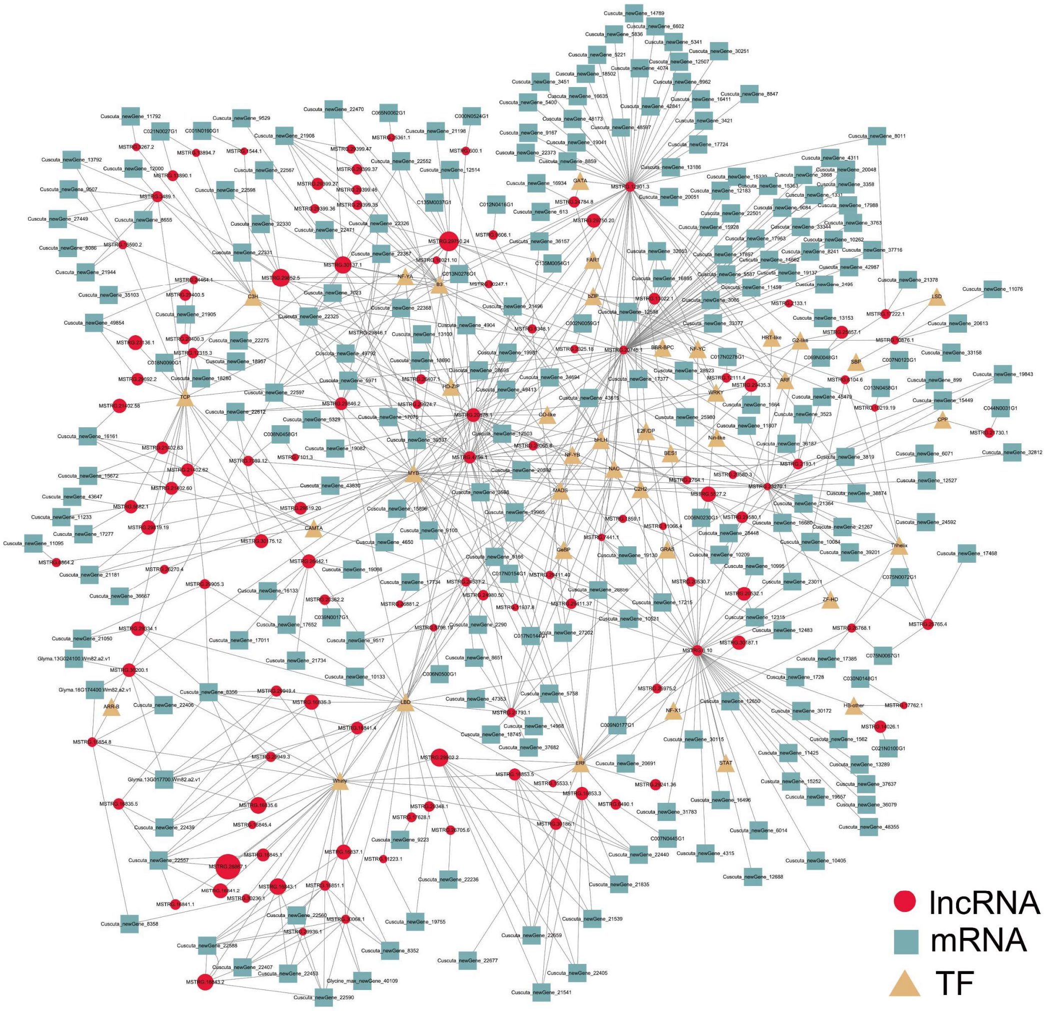

Figure S8. Representative predicted network with interconnected members among mobile lncRNAs (red circle), mobile lncRNAs target mRNAs (blue square), and transcription factors (yellow triangle). The size of the circle is positively correlated with the expression of lncRNAs.
